# Adhesive systems: important aspects related to their composition and clinical use

**DOI:** 10.1590/S1678-77572010000300002

**Published:** 2010

**Authors:** Mario Honorato SILVA E SOUZA JUNIOR, Karina Gama Kato CARNEIRO, Marcelo Figueiredo LOBATO, Patrícia de Almeida Rodrigues SILVA E SOUZA, Mário Fernando de GÓES

**Affiliations:** 1 DDS, MSc, PhD Associate Professor, Department of Restorative Dentistry, Dental School, Federal University of Pará, Belém, PA, Brazil.; 2 DDS, Graduate student, Dental School, Federal University of Pará, Belém, PA, Brazil.; 3 DDS, MSc, PhD Associate Professor, Department of Endodontics, Dental School, Federal University of Pará, Belém, PA, Brazil.; 4 DDS, MSc, PhD Associate Professor, Department of Restorative Dentistry, Piracicaba Dental School, State University of Campinas, Piracicaba, SP, Brazil.

**Keywords:** Dentin-bonding agents, Dentin, Dental adhesives, Chemical composition

## Abstract

This literature review article addresses the types and the main components of different etch-and-rinse and self-etch adhesive systems available in the market, and relates them to their function, possible chemical interactions and influence of handling characteristics. Scanning electron microscopy (SEM) images are presented to characterize the interface between adhesives and dentin. Adhesive systems have been recently classified according to their adhesion approaches in etch-and-rinse, self-etch and glass ionomer. The etch-andrinse systems require a specific acid-etch procedure and may be performed in two or three steps. Self-etch systems employ acidic monomers that demineralize and impregnate dental substrates almost at the same time. These systems are separated in one or two steps. Some advantages and deficiencies were noted for etch-and-rinse and self-etch approaches, mainly for the simplified ones due to some chemical associations and interactions. The SeM micrographs illustrate different relationships between adhesive systems and dental structures, particularly dentin. The knowledge of composition, characteristics and mechanisms of adhesion of each adhesive system is of fundamental importance to permit the adoption of ideal bonding strategies under clinical conditions.

## INTRODUCTION

Throughout the last decades adhesive systems have received different classifications, generally based on modifications in their compositions. These practices led to several complex and confusing classifications that have brought some difficulties to clinicians for selection and use of dental adhesives. Van Meerbeek, et al.^39^ (2003) proposed a simple classification based on the interaction of adhesives with dental substrates and number of steps: etch-and-rinse (two- and three-step adhesives), self-etch (one- and two-step adhesives) and glass ionomer. All of them have received important modifications in the last years. These modifications were made based on the increasing of knowledge of their compositions and adhesion mechanisms.

Indeed, the best understanding of the role of dental substrates in the adhesion process has helped researchers and manufacturers developing and improving dental adhesion.

This literature review article addresses the types and the main components of different etch-andrinse and self-etch adhesive systems available in the market, and relates them to their function, possible chemical interactions and influence of handling characteristics.

### Etch-and-rinse ADHESIVE systems

Etch-and-rinse adhesive systems can be either three- or two-step materials depending on whether primer and bonding are separated or combined in a single bottle. The adhesion strategy involves at least two steps and, in its most conventional form, three steps with successive application of the conditioner (acid etchant), followed by the primer (adhesion promoting agent), and eventually, application of the bonding agent (adhesive resin). The simplified two-step version combines the second (priming) and third (bonding) steps, but still follows a separated etch and rinse phase^2,9,39^. Figure 1 describes the sequence of procedures of etch-and-rinse systems.

**Figure 1:** Etch-and-rinse adhesive systems - adhesion strategies according to the number of steps

**Number of steps**	**Adhesion strategy**	
Three-step	Acid-etching	Priming Bonding
Two-step	Acid-etching	Priming and bonding

### Acid Conditioning

**Figure 2:**
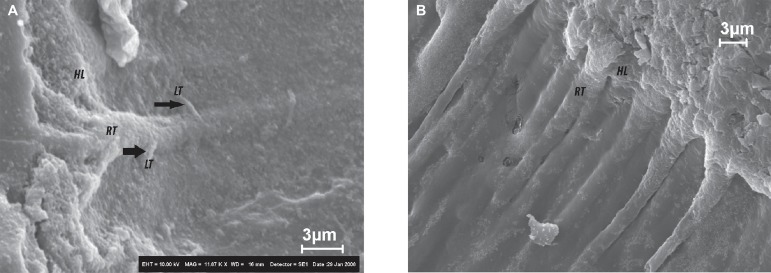
SEM images of dentin-adhesive interfaces. A - Hibrid layer (HL) formed in dentin after use of XP Bond (Dentsply) two-step etch-and-rinse system. Elongated, funnel-shaped resin tags (RT) can be seen due to the demineralization produced by phosphoric acid etching. Lateral tubules (LT and black arrows) were also filled with resin, important structures in the adhesive mechanism. B - This SEM image was obtained using All Bond 3 (Bisco) three-step etch-and-rinse system. It is also possible to see the hybrid layer (HL) and long, funnel shaped resin tags (RT)

Acid-etching of enamel is a widely accepted clinical procedure due to its chemical structure and has increased the life of composite resin restorations by decreasing the possibility of marginal staining, secondary caries and postoperative sensitivity^19^. The effects of conditioning procedure may vary widely, depending on several factors, such as type (sound or sclerotic), depth and tubule orientation^7,20,41^. Some aspects of the conditioned/primed area however are the same. The tubule access becomes funnel shaped and the resin tags are normally elongated. These aspects can be seen in Figures 2A and 2B. Ideally, acid etching with 35% H_3_PO_4_ should not exceed 15 s. Prolonged acid application may lead to structural modification of the exposed collagen^3^.

### Monomers

In the two-step systems the hydrophilic and hydrophobic monomers are combined with solvent(s) in the same bottle. These associations may cause some chemical disorder during clinical application. The presence of unprotected dentin collagen fibers may be explained by the presence of residual water that may prevent complete monomer infiltration in the deep demineralized zone, which compromises ideal adhesive infiltration and polymerization^16,27^. These factors could be responsible for the degradation of resin-dentin interfaces over short periods of time. The instability of bonds over longer time periods has been attributed to the degradation of both exposed collagen and resin monomers^10,16^.

**Figure 3:**
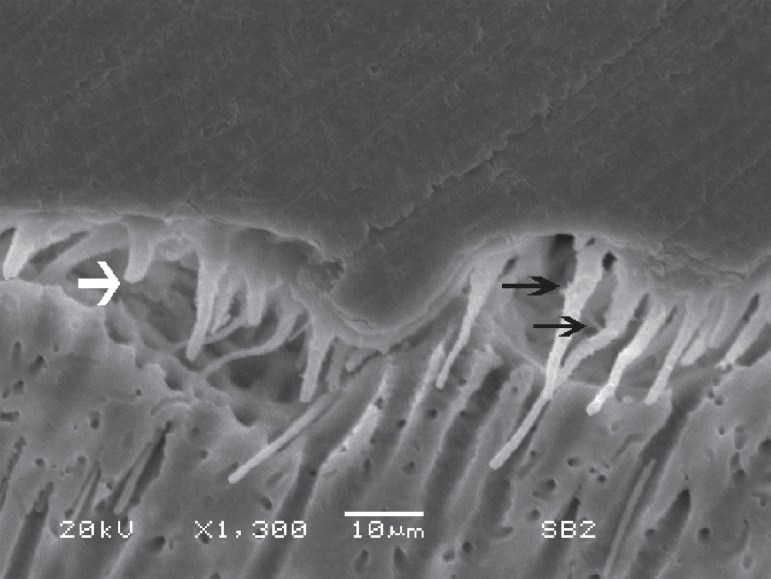
An adhesive defect can be seen in this image. Several reasons may contribute to this event and polymerization deficiency due to the excess of residual solvent may be one of them. The resin tags seem to have detached from the dentinal tubules, even in a presence of some small lateral resin projections (black arrows). Only few tags appear to be broken (white arrow)

HEMA (2-hydroxymethyl methacrylate) is a very popular monomer which is in widespread use^17^. It is much employed either in three- and two-step etch-and-rinse systems and one reason for this preference is related to its hydrophilicity that makes it an excellent adhesion promoter enhancing bond strength^11,36,39^. On the other hand, the hydrophilic characteristic may, in uncured and cured states, readily absorb water^36^. In the uncured state the absorption of water may lead to dilution of the monomer to the extent that polymerization is inhibited^15^ , compromising the initial bond strength, which may cause adhesion breakdown (Figure 3). This aspect must be especially considered during clinical procedure when HEMA-rich adhesives have their polymerization delayed. After polymerization HEMA will still exhibit hydrophilic properties. Considering its permeable structure, water treeing will be prone to occur^29,32^. The presence of water within hybrid layer may cause hydrolysis, a chemical process that breaks covalent bonds between polymers by addition of water on ester bonds, resulting in resin degradation compromising bond strength in latter periods of time^30^. Since simplified (two-step) etch and rinse adhesives contain higher percentages of hydrophilic monomers compared to three-step adhesive^28^ , they exhibit greater permeability after polymerization, thus facilitating the presence of water-filled areas within hybrid layer^29^. Recently, it can be noted the trend towards decreasing the amount of strong hydrophilic monomers, such as HEMA, and replacing this portion by UDMA or TEGDMA^36^.

**Figure 4:**
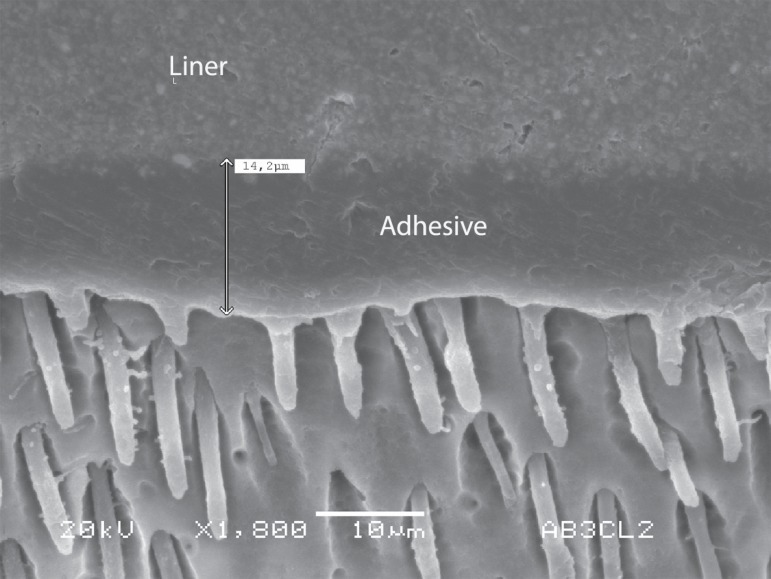
A hydrophobic coat (liner) can been used on the top of the hydrophilic primed dentin in some adhesive systems, such as All Bond 3

The hydrophobic coat of the three-step etchand-rinse system may in part overcome the water movement throughout the bonded interface. In areas of hybrid layer defects the passage of fluids speeds up during biting and temperature shifts^7^. This passage may occur in different directions, from and towards the pulp, and from and towards the oral environment. Thus, it would be feasible, in a presence of a hydrophobic coat, a reduction of this movement, preserving adhesive interface from hydrolysis and also decreasing sensitivity^7^. Besides its hydrophobic nature, the higher degree of polymerization of the nonsolvated hydrophobic agents was correlated to less permeability to water^5^. Some newer adhesive systems, such as All Bond 3 (Bisco Inc., Schaumburg, IL, USA), renamed this hydrophobic sealer as liner (Figure 4).

### Solvents

A very important component of the adhesive systems is the solvent. The low viscosity of primers and primer-adhesive resins is partially due to the dissolution of monomers in a solvent. This association will improve the diffusion ability in the porous conditioned substrate, especially in dentin due to its hydrophilic nature. In adhesives, water, ethanol and acetone are the most commonly used solvents.

As mentioned above, solvents are important to assure the diffusion of monomers into the demineralized dentin. After diffusion the solvents must be eliminated from adhesive, otherwise remaining solvent in the adhesive may jeopardize polymerization due to the dilution of monomers and may result in voids and increase the permeability of the adhesive layer^12,15^. Effect of evaporation of the primer components is important to ultimate tensile strength of primer-adhesive mixture. Complete evaporation is however difficult to achieve because it is limited to the short clinical time^12^. The evaporation of the solvent is related to its vapor pressure. Higher vapor pressure of the solvent implies faster evaporation^21^. While the solvent evaporates, the solvent-monomer ratio decreases, as well as the vapor pressure. Thus, within the clinical time, residual solvent may remain in the adhesive and the consequences are directly related to its amount^7^.

**Figure 5:**
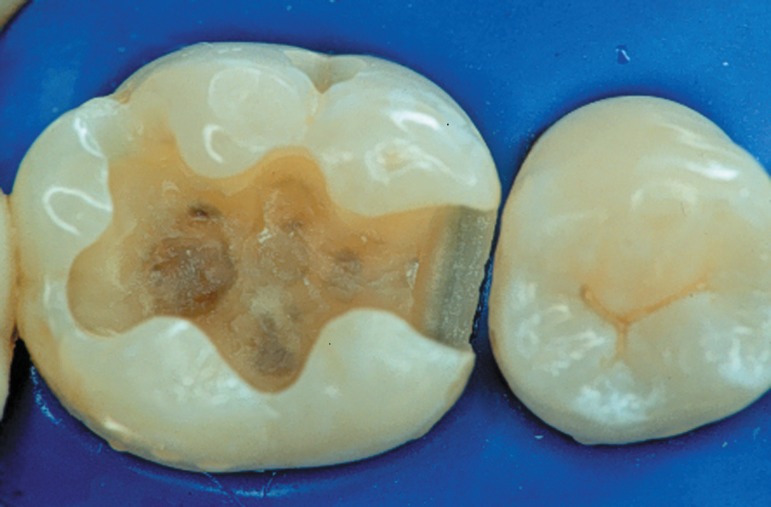
Dental surface aspect after acid-etching when a water-based system is used. The surface must be moist but with no visible shiny appearance. The blot-dry technique helps achieving this condition

**Figure 6:**
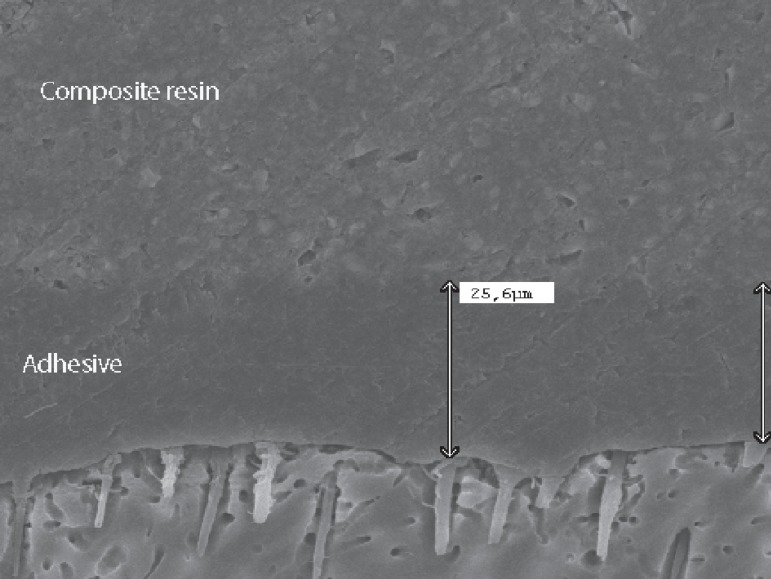
Thickening effect (24.6 μm) probably due to the application of an extra coat of a water-ethanol based adhesive system (Single Bond)

The application technique is different depending on the solvent. Water is a poor solvent for organic compounds (such as monomers). This difficulty can be overcome by addition of a secondary solvent, such as ethanol and acetone (azeotrope)^36^. As the vapor pressure is lower, water-based adhesive, takes longer to evaporate. Therefore, it will need more clinical time to help the monomer diffusion. A rubbing application technique is also cooperative to assure both monomer diffusion and solvent evaporation as well^7^. As this type of adhesive already contains water, the amount of water in the substrate must not be excessive. The dental substrate, especially dentin, must be moist, but with no visible shine^7^ on the surface (Figure 5). One advantage of these agents is their capacity of expanding collagen, in case of substrate overdrying (excessive air blowing)^21^. Normally one coat of a material in this category is sufficient to appropriately cover the entire surface. extra coats may have a thickening effect and result in the imprisonment of solvent between layers (Figure 6). This may lead to lower bond strength values^36^.

**Figure 7:**
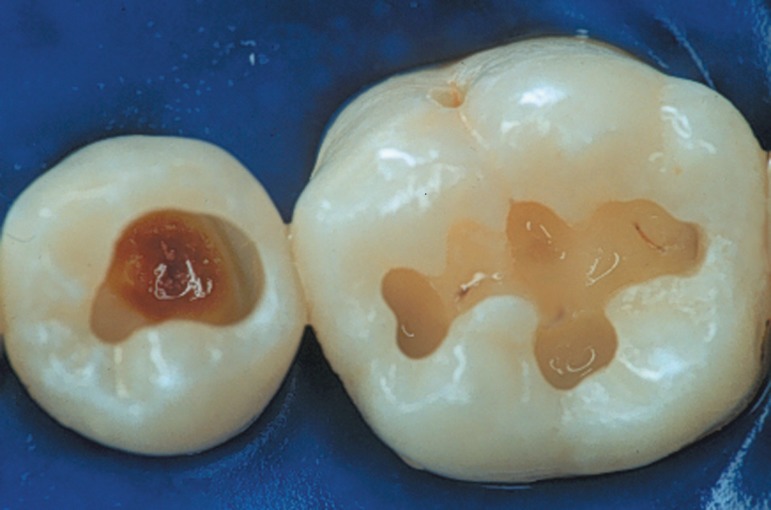
Different condition compared to the image shown in Figure 5 is the surface aspect of dental substrates. It is possible to see a moist and shiny appearance, surface state appropriate to receive acetone-based (no water) systems

**Figure 8:**
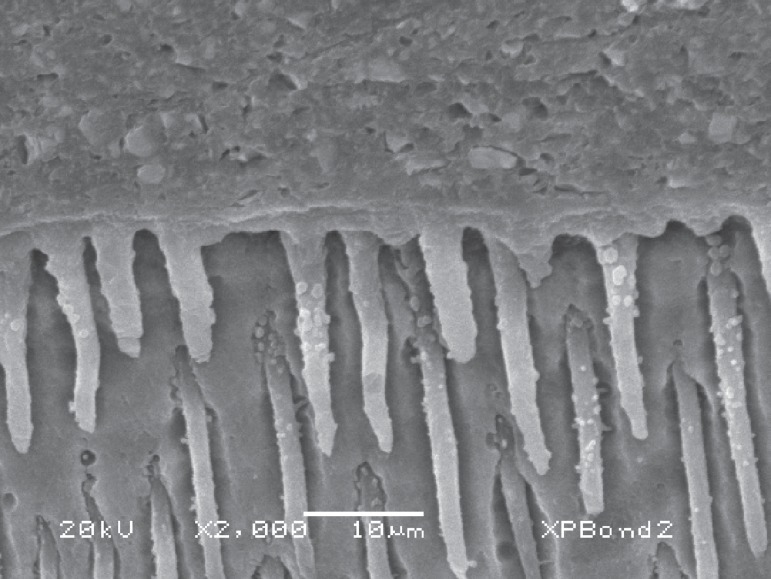
A thick layer of adhesive can be seen when XP Bond (T-Butanol solvent) was applied. It is also possible to see some filler particles on the resin tags

On the other hand, with acetone-based adhesives (without water), as the vapor pressures of these solvents are much higher, the primer or primer-adhesive must be left undisturbed on the surface and the substrate must be moist with a shiny appearance^7^ (Figure 7). These systems will not be able to re-expand the collapsed collagen, on an over dried dentin surface, thus avoiding correct monomer diffusion. Figure 8 depicts an adhesive interface prepared with the XP Bond (Dentsply, De Trey, Konstanz, Germany), which employs the T-butanol solvent. The thickness of the adhesive coat is very thin compared to those obtained with All Bond 3 (Bisco Inc., Schaumburg, IL, USA) (Figure 4) and Single Bond (3M/ESPE St. Paul, MN, USA) (Figure 7), which uses a water-ethanol mixture.

## SELF-ETCH ADHESIVE SYSTEMS

The self-etch approach is an alternative based on the use of non-rinse acidic monomers that simultaneously condition and prime tooth tissues. Regarding technique-sensitivity, this approach seems clinically most promising, since it eliminates the rinsing phase, which not only reduces clinical application time, but significantly decreases the technique sensitivity or the possibility of making errors during application^1,2,8,39^. Another important characteristic of the self-etch approach is that infiltration of monomers occurs simultaneously with the self-etch process; therefore, the possibility of discrepancies between both processes^39^ and consequently the presence of an unprotected collagen fibers area is significantly reduced, as is the nanoleakage^2,9,29^. Figure 9 depicts some characteristics and the adhesion strategies of self-etch systems.

**Figure 9:** Self-etching systems - adhesion strategies according to the number of steps

**Number of steps**	**Adhesion strategy**
Two-step (2 bottles)	Etching / priming Bonding
One-step - pre-mixing required (2 bottles)	Etching / priming/bonding
One-step - no mixing required (1 bottle)	Etching / priming/bonding

### Aggressiveness of self-etch systems

**Figure 10:**
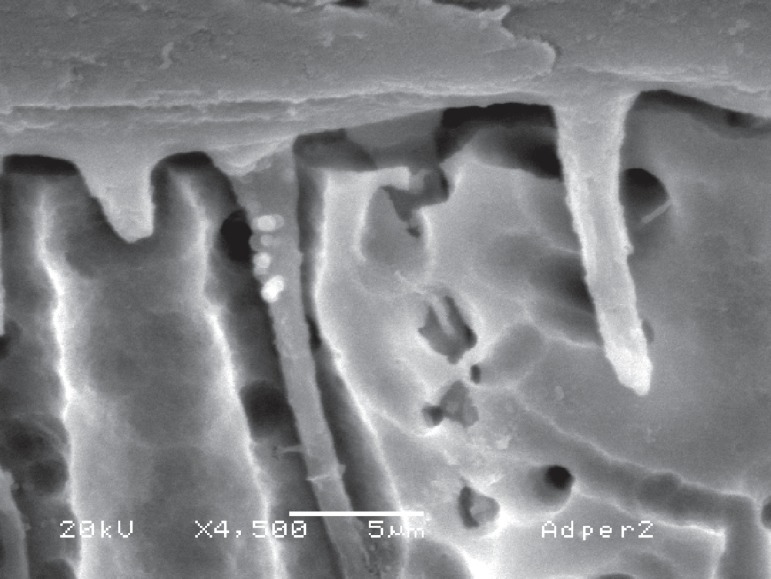
The strong self-etching systems may result in long and funnel-shaped resin tags due to their aggressiveness. This image was obtained using Adper Prompt L-Pop, which has a pH of 0.8

**Figure 11:**
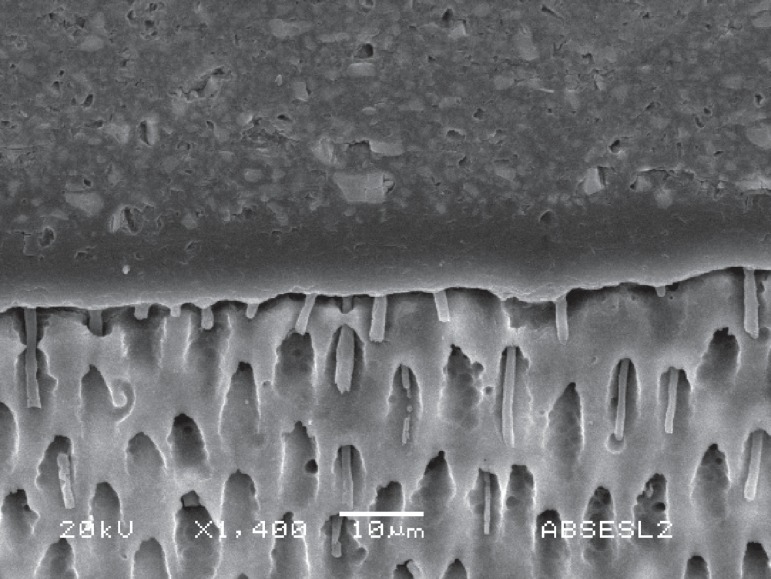
When a mild self-etching agent such as All Bond SE (pH 2.2) is used, short and cylindrical resin tags are produced

**Figure 12:**
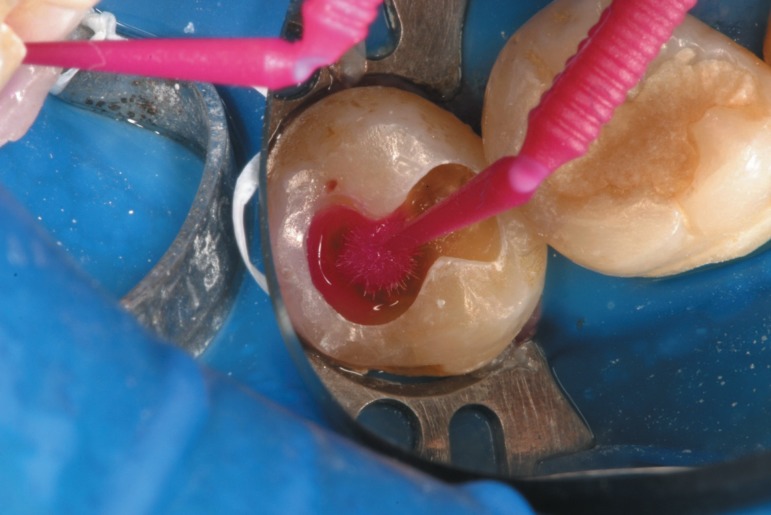
Liquid A of Adper Scotchbond SE is first applied to the cavity. Water is the main compound of this part of the system

**Figure 13:**
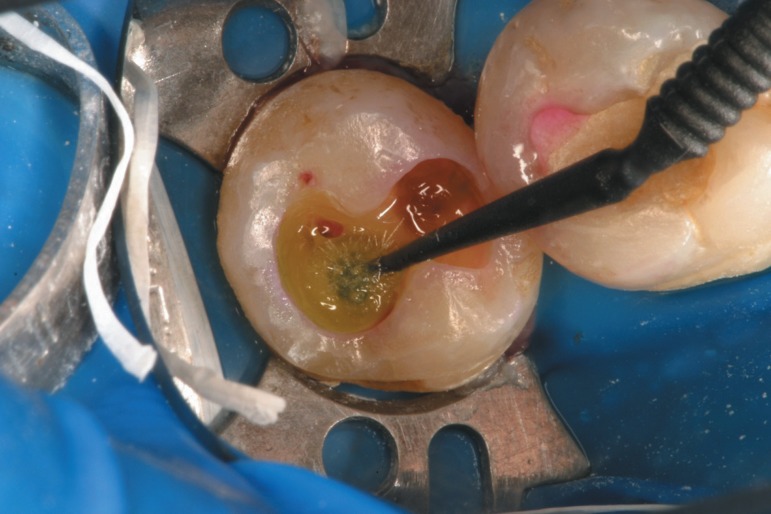
When liquid B, an association of monomers, filler and initiator, is transferred in to the cavity, the acid reaction takes place. The operator needs to mix both liquids (A and B) inside the cavity. The pink appearance starts fading

**Figure 14:**
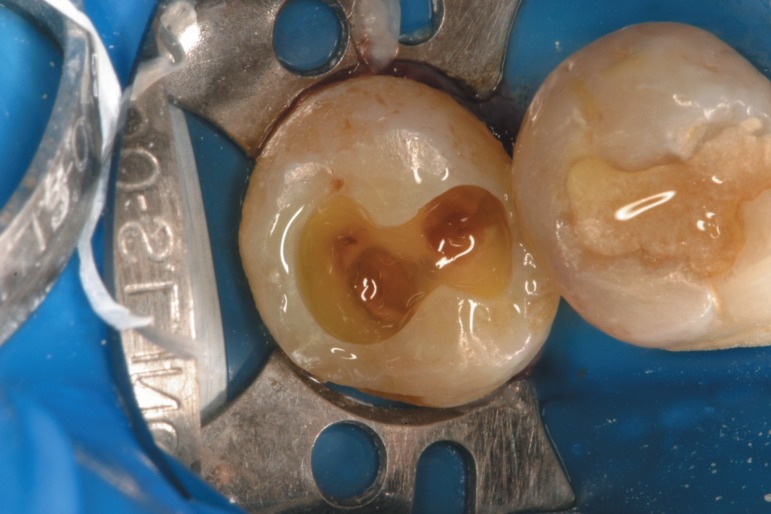
A yellowish appearance indicates that all the compounds were mixed. At this stage water and residual monomers must evaporate before polymerization

Some questions however arise from this particular approach: 1- Could the presence of dissolved hydroxyapatite and residual smear layer remnants interfere in the bond? 2- Are the self-etch systems able to properly demineralize enamel or sclerotic dentin? The self-etch systems were gradually modified in the last few years and one important change was the increase in their aggressiveness^35^. Depending on etching aggressiveness, self-etch adhesives can be subdivided into strong (pH≤1), intermediary strong (pH≈1.5) and mild (pH≈2.0)^35,36,39^. Strong self-etch adhesives present higher acidity compared with mild and intermediary strong systems and the interaction patterns observed in enamel and dentin resembles a phosphoric acid treatment after etch and rinse approach^22^. Figure 10 depicts resin tags formation after the use of Adper Prompt L-Pop (3M/eSPe, St. Paul, MN, USA), pH-0.8. The tubules are wide open, the resin tags have a funnel shape and are elongated. On the other hand, dentin treated with a mild self-etch system (All Bond Se; Bisco Inc., Schaumburg, IL, USA), pH 2.2, exhibits cylindrical and short tags (Figure 11). Despite the similar etching pattern with the etch and rinse systems, the bond strength observed for the strong self-etch adhesives was lower, especially at dentin^38, 39^. The presence of water in the composition of self-etch systems is necessary to trigger the demineralization process. The excess of residual water during polymerization may be one of the reasons for the poor bond strength^7, 9, 18, 39^. Indeed, adhesive systems that contain high concentrations of acidic resin monomers behave like permeable membranes^29^ and allow water movement from dentin to the composite-adhesive interface^31^. This may further compromise the durability of resin-dentine bonds and affect the coupling of the simplified adhesives to auto-cured (or dual-cured) composites^7, 30^. These two aspects; low initial bond strength and gradual degradation, due to hydrolysis, have made researchers and manufacturers rethink about monomers, pH and association of components in the bottles. Some newer self-etch adhesives present higher pH, such as Xeno IV (Dentsply Caulk, Milford, DE, USA), pH 2.1 and All Bond SE (Bisco Inc., Schaumburg, IL, USA), pH 2.2. Others, such as Adper SE Plus (3M/ ESPE, St. Paul, MN, USA) present a very low pH (<1), their components are strategically distributed in the bottles. Liquid A formed of water, HEMA and a pink dye is first applied onto the cavity (Figure 12). The water will only meet the monomers in a second step, when liquid B is transferred to the cavity (Figure 13). A continuous brushing procedure is advised to force the contact of all components and help the evaporation of excess water. The pink appearance starts fading immediately and a light yellowish look takes place (Figure 14). Conversely, the Adper easy One Bond (3M/ESPE, St. Paul, MN, USA) has all the components associated in the same bottle, including water. However, the pH of the mixture is much higher (2.3) than the pH of Adper SE Plus (3M/ESPE, St. Paul, MN, USA). These differences imply in distinct application techniques and storage. While easy One Bond must be kept under refrigeration, Adper Scotchbond SE (3M/ ESPE, St. Paul, MN, USA) can be maintained at room temperature.

### Monomers

One significant advantage of a mild self-etch system is to keep some hydroxyapatite crystal around collagen fibers^39^. This characteristic may protect the collagen against hydrolysis and, thus, early degradation of the bond^25^. The adaptation of etch-and-rinse adhesives to denuded collagen fibrils has been considered poor^39^ , and hence a possibility of chemical interaction between residual hydroxyapatite and functional monomers is expected to improve bonding. Researchers have pointed out that some functional monomers in self-etch adhesives, such as 10-MDP present in the Clearfil Liner Bond 2 and SE Bond (Kuraray Medical Inc., Tokyo, Japan), 4-MET as part of the Unifil Bond and G-Bond (GC, Tokyo, Japan) and phenyl-P found in the Clearfil Liner Bond 2 (Kuraray Medical Inc., Tokyo, Japan), can chemically interact with hydroxyapatite within a clinical time, and this interaction has been connected to better resistance towards degradation by prevention of micro and nanoleakage^39, 42^. The strong one-step self-etch adhesive Prompt-L-Pop (3M/ESPE, St. Paul, MN, USA), performed very poorly, with a retention rate of 65% after one year^2^. On the other hand, mild two-step self-etch adhesive Clearfil SE Bond (Kuraray Medical Inc, Tokyo, Japan), which contain the 10-MDP, exhibited excellent result for up to two years^33^.

Recently, Ikeda et al.^13^ (2008), evaluated the effect of air-drying on the ultimate microtensile bond strength of HEMA-rich and HEMA-free one-step adhesives. It could be shown that the evaporation degree of residual monomers and solvents increased with the extension of air-drying. Longer air-drying time (10 sec.) resulted in a statistically significant higher microtensile bond strength for the HEMA-rich (Clearfil S Bond/ Kuraray Medical Inc, Tokyo, Japan) compared to the HeMA-free (I Bond/ Hereaus-Kulzer, Hanau, Germany and G-Bond/ GC, Tokyo, Japan). HeMA has been used as adhesion promoter in most of the self-etch systems due to some of its characteristics already mentioned^11, 36^ , however, high concentrations in the adhesive composition, normally present in the one-step self-etch adhesives may have immediate (lower bond strength) and posterior (hydrolysis) deteriorating effects on the mechanical properties of the resulting polymer^13, 15, 29, 32, 36^.

The effects of the amount of HEMA on initial bond strength and deteriorating effects of hydrolysis are somehow correlated. Higher concentration of HEMA (19-36%)^38^ in the composition of one-step self-etch adhesives may reduce initial bond strength (particularly 36%) due to the attraction of water and the presence of droplets on dentin, especially after delay-curing the composite. This water may contribute also to monomers dilution and reduction on polymerization degree^15, 36, 38^. Higher bond strengths were determined at 10% of HeMA in the composition of some experimental adhesives formulations^38^.

A possibility to prevent hydrolysis of hydrophilic monomers, such as HEMA, present in high concentrations in some simplified self-etch systems, is to coat the primed dentin with additional layer of hydrophobic agent onto the polymerized one-step adhesive agent, converting them in a two-step system^4,7,16,26^.

In the last few years, some researchers have proposed the use of monomers with different hydrophilic levels on dentin. Nishitani, et al.^18^ (2006), examined the microtensile bond strength of five experimental adhesives (50wt%ethanol/50%comonomers) of various degree of hydrophilicity to acid etch dentin that was left moist with water or ethanol, or air-dried. Following the composite resin application, specimens were prepared for microtensile test. For all three types of dentin treatments, higher bond strengths were achieved with increased resin hydrophilicity. The lowest bond strengths were obtained on air-dried dentin, while the highest ones were achieved when dentin was bonded moist with ethanol. Wet-bonding with ethanol achieved higher bond strengths with hydrophobic resins than were possible with water-saturated dentin. These observations open possibilities to use less hydrophilic monomers on dentin bonding with the purpose of reduce the deterioration potential of some adhesives by hydrolysis^18^.

### Solvents

As mentioned before, water is an indispensable component of self-etch agents, in order to ionize the acidic monomers and trigger the demineralization process^7,22,36,39^. The strong self-etch agents are likely to contain higher amounts of water. A concern is the effect of residual water that remains within the adhesive interface, which hardly can be completely removed^39^. Some self-etch agents present only water as solvent, such as Adper Se Plus (3M/ESPE, St. Paul, MN, USA), AdheSe (Ivoclar Vivadent, Schaan, Liechtenstein), Adper Prompt (3M/ESPE, St. Paul, MN, USA). However, in many systems, the water is associated to ethanol, acetone or even to monomers, such as the N,N-diethanol p-toluidine, present in the Clearfil SE Bond (Kuraray Medical Inc., Tokyo, Japan) adhesive. Special attention should be directed to water-based agents, mainly the all-in-one agents. A multiple layer application under a continuous brushing technique has also been claimed to increase the bond strength of these materials^14,23^. On the other hand, as water has been related to phase-separation, polymerization-inhibition and reduced shelf-life, the development of self-etch water-free adhesive has been already proposed^37^. The water necessary to trigger the acidic reactions would come from the dental substrate.

Another simple approach to improve bonding efficacy and stability is correlated with enhanced solvent evaporation. The air-blowing of the adhesive might help to remove interfacial water, thus improving bonding effectiveness^13, 34^. However, this procedure is somehow controversial^40^ because it has been stated that a strong air stream may increase the adhesive thickness in the cavity angles and denude part of dentin. It is an important issue related to cavity geometry, normally different from the flat dental surface used for bond strength tests. A mild and extended air-blow should, however, be cooperative to the evaporation of solvent and residual monomers.

## Final Considerations

The research field of dental adhesion is wide open. Several research lines have been proposed and investigated in the last years. The use of an extended polymerization time^5^ , the application of electrical current to dental adhesives^4,24^ , the relevance of matrix metalloproteinase inhibitors^6^ , the development of water-free adhesives^37^ , and the use of hydrophobic monomers in conditioned dentin treated with ethanol solutions^18^ , for instance, are some of the innumerous possibilities under investigations. Also, the balance among some essential components such as monomers and solvents has been studied^38^. As it could be noted by the issues discussed in this literature review, keeping an updated knowledge of the composition, characteristics and mechanisms of adhesion of the currently available adhesive systems as well as knowing how the dental substrates interact with these materials are essential to achieve the best results in adhesion.
